# The rcdk and cluster R packages applied to drug candidate selection

**DOI:** 10.1186/s13321-019-0405-0

**Published:** 2020-01-20

**Authors:** Adrian Voicu, Narcis Duteanu, Mirela Voicu, Daliborca Vlad, Victor Dumitrascu

**Affiliations:** 1 Department of Medical Informatics and Biostatistics, Victor Babes University of Medicine and Pharmacy, E. Murgu 2, 300041 Timisoara, Romania; 2 Dep. CAICAM, Politehnica University of Timisoara, Pirvan Boulevard 6, Timisoara, Romania; 3 Department of Pharmacology-Clinical Pharmacy, Victor Babes University of Medicine and Pharmacy, E. Murgu 2, 300041 Timisoara, Romania; 4 Department of Pharmacology, Victor Babes University of Medicine and Pharmacy, E. Murgu 2, 300041 Timisoara, Romania

**Keywords:** Cytostatic, Molecular fingerprint, Rcdk, Clusters

## Abstract

The aim of this article is to show how thevpower of statistics and cheminformatics can be combined, in R, using two packages: *rcdk* and *cluster*.

We describe the role of clustering methods for identifying similar structures in a group of 23 molecules according to their fingerprints. The most commonly used method is to group the molecules using a “score” obtained by measuring the average distance between them. This score reflects the similarity/non-similarity between compounds and helps us identify active or potentially toxic substances through predictive studies.

Clustering is the process by which the common characteristics of a particular class of compounds are identified. For clustering applications, we are generally measure the molecular fingerprint similarity with the Tanimoto coefficient. Based on the molecular fingerprints, we calculated the molecular distances between the methotrexate molecule and the other 23 molecules in the group, and organized them into a matrix. According to the molecular distances and Ward ’s method, the molecules were grouped into 3 clusters. We can presume structural similarity between the compounds and their locations in the cluster map. Because only 5 molecules were included in the methotrexate cluster, we considered that they might have similar properties and might be further tested as potential drug candidates.

## Introduction

Discovery, synthesis and production of new drugs is still challenging for researchers because of the complex structures of endogenous molecules involved in the pathogenesis of diseases such as AIDS, cancer and autism [16]. Modern drug research is characterized by the growing number of lead molecules and the need to examine and characterize all of these compounds over short periods [14, 39].

Chemical database mining based on the similar compounds search is an in silico method widely used in the drug discovery process [28, 33]. It can be used in the initial stages of drug discovery and speeds up the entire process [10]. The requirement to store, manage and analyse these rapidly growing resources has given rise to a relatively new field known as computer-assisted drug design (CADD) [22, 39, 40].

Computational chemistry is a very effective approach in drug design for the identification of lead compounds. Various virtual screening techniques can be used to reduce the cost and time required to identify a potential drug [2]. As a computational method in drug discovery and virtual screening, clustering of chemical compounds by the similarity of their molecular fingerprints can be used to identify similar structures in a large set of similar data [38, 41]. Their virtual screening performance is comparable to other,more complex, methods. There are many types of fingerprints, each of which represents a different aspect of the molecule [37, 42, 43].

Clustering is an unsupervised machine learning technique that groups data with similar properties. This technique for statistical data analysis is widely used in cheminformatics [19].

A *cluster* is, in this case, a collection of molecules which are organized in groups, according to their molecular fingerprints [3, 17].

Despite the large number of clustering methods, only a few of them are widely used in practice. In this paper, only two of them were used, which proved to be suitable for chemical structure analysis: hierarchical clustering and K-means method. Regardless of the method, the results were the same.

## Methods

All the software used for this article can be installed on Windows, Linux or macOS operating systems.

Initially, built as an environment for statistical computing, R, a GNU project, provides a wide variety of packages for cheminformatics that are suitable for calculating molecular fingerprints and clustering [13, 25, 46]. The latest version of R can be downloaded form the CRAN repository. R Studio is considered one of the best IDEs (integrated development environments) for R and was also used for this article. In this paper, R version 3.6.0 and RStudio version 1.2.1335 were used.

Marvin Sketch version 17.3, from ChemAxon, an academic software package, was used to draw, display and characterize the chemical structures [8]. The molecules were imported in Marvin Sketch using their IUPAC names (International Union of Pure and Applied Chemistry) and then saved as SMILES and SDF formats [31]. Then, they were imported and processed in R [12, 28].

### R applications for cheminformatics and computational chemistry

Its flexibility and wide application fields have made the R programming environment a popular choice in a large number of areas.

In the field of cheminformatics, R offers several tools that are able to treat a large variety of issues related to the statistical modelling of chemical information. The *rcdk* package, version: 3.4.7.1, used in the present work, provides direct access from the R environment to the CDK (Chemistry Development Kit), a powerful Java framework for cheminformatics [6, 47].

CDK is a collection of free Java libraries that supports a wide variety of cheminformatics functionality. This platform allows us to read different molecular formats, calculate molecular descriptors and evaluate molecular fingerprints.

The cluster package, version 2.1.0, can be used to find groups of molecules that share similar chemical properties [2, 23].

The packages can be installed using the function "install.packages()". The general syntax is listed below: 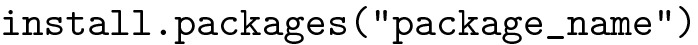


To use a package, it must be loaded in the R environment using the function ** library().**

In addition to *rcdk*, some other packages were also needed: 
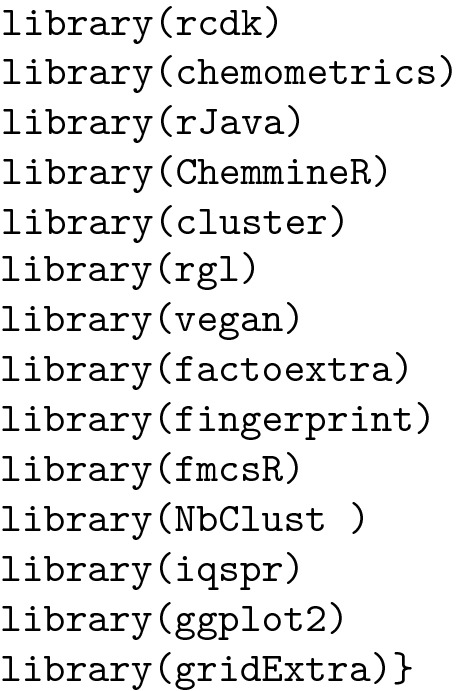


#### Importing and viewing the "drug candidate" molecules in R

In order to manipulate the chemical structures in R, we assigned them a code, starting with CMP1 for methotrexate and ending with CMP24 for the last structure. The Methotrexate molecule (coded as "CMP1") was downloaded from ZINC15, a free database of commercially available compounds, in both SMILE and SDF file format.

In SDF format, the molecule of methotrexate can be imported and visualized in R using the code listed below: [13, 44] 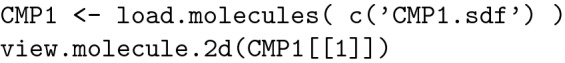


**Fig. 1:**
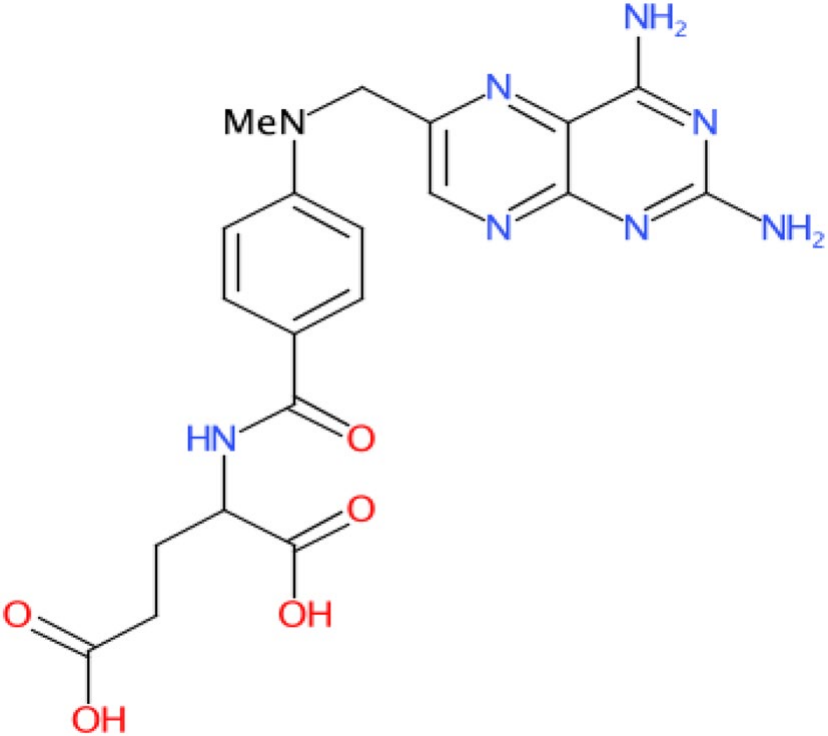
Methotrexate molecule visualisation in R

The result is depicted in Fig. 1:

All the molecules were imported in SDF format and visualized in R as a grid. 
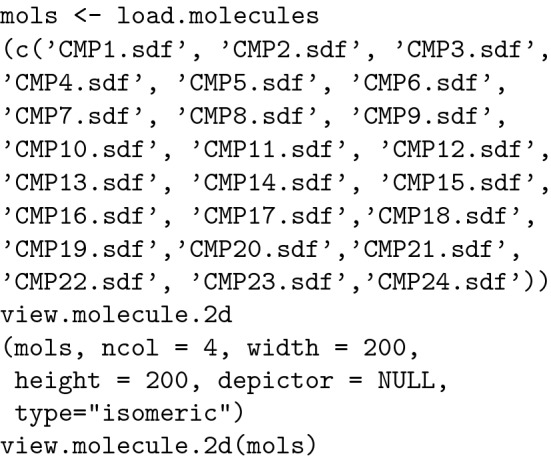


**Fig. 2:**
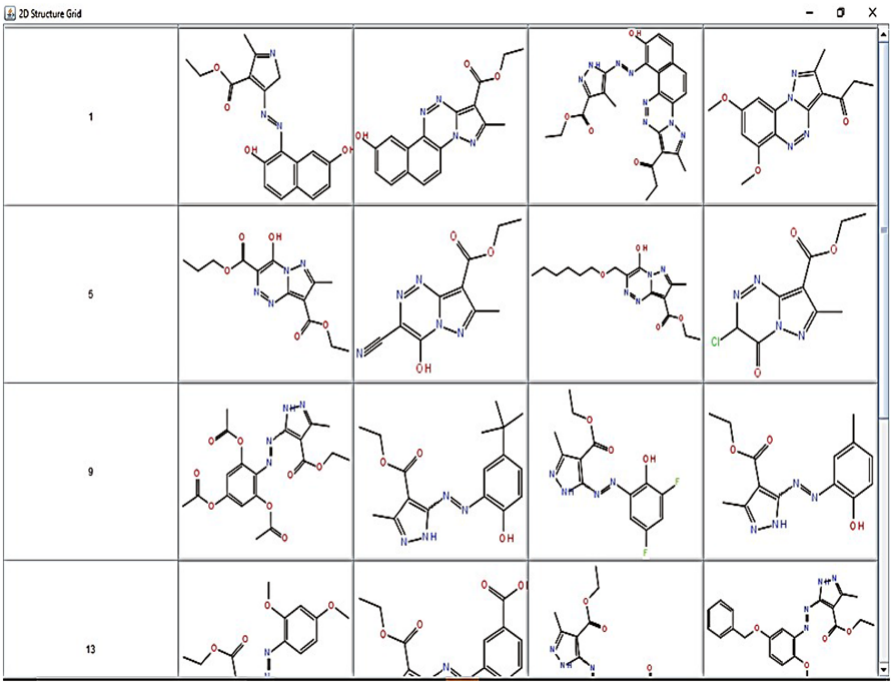
Molecule set visualization

The result is depicted in Fig. 2:

#### Computation of the molecular descriptors (physicochemical properties of the molecules)

The *rcdk* package can also be used to calculate a set of physicochemical properties of the molecules:

**The number of atoms:** 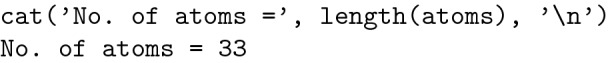


**The number of chemical bonds:** 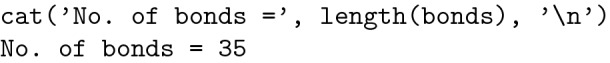


**The coordinates of the first atom:** 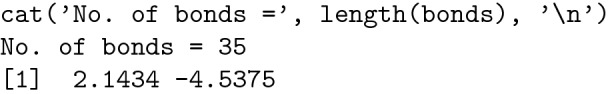


It is also possible to calculate the coordinates for all the atoms present in the molecule: 
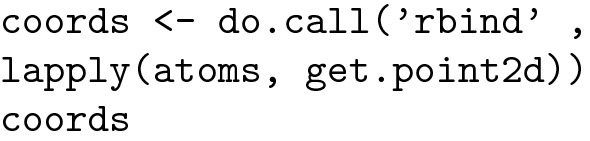


R can compute a set of molecular descriptors, grouped into 5 different categories: 
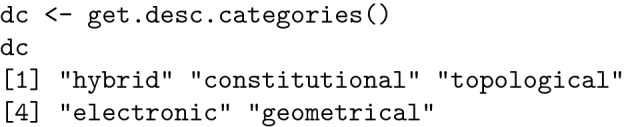


Category 2 (constitutional), important in QSAR, contains 15 descriptors, which are listed below: [14]. 
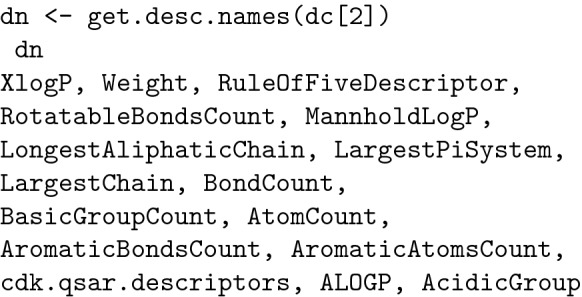


Regarding drug design, the evaluation of AlogP is given a higher importance than that of other descriptors: 
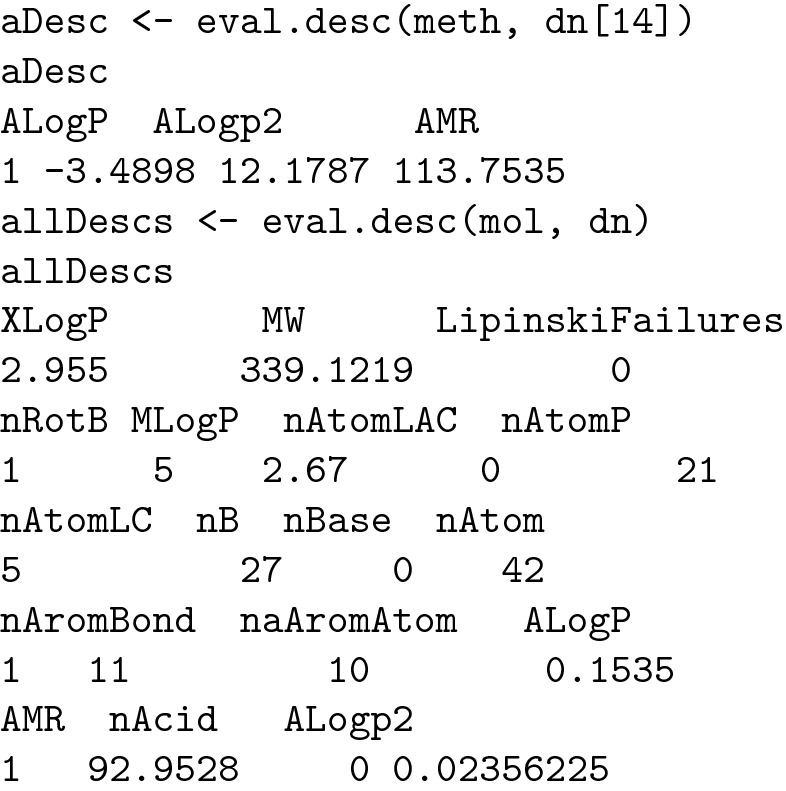


#### Computation of the molecular fingerprints

Molecular fingerprints can be computed by several methods, but in the case of aromatic compounds the "extended" method is preferred. "Extended" fingerprints have a length (the number of bits ) of 1024, compared to 166 for "maccs "type fingerprints [11, 24, 36]. 
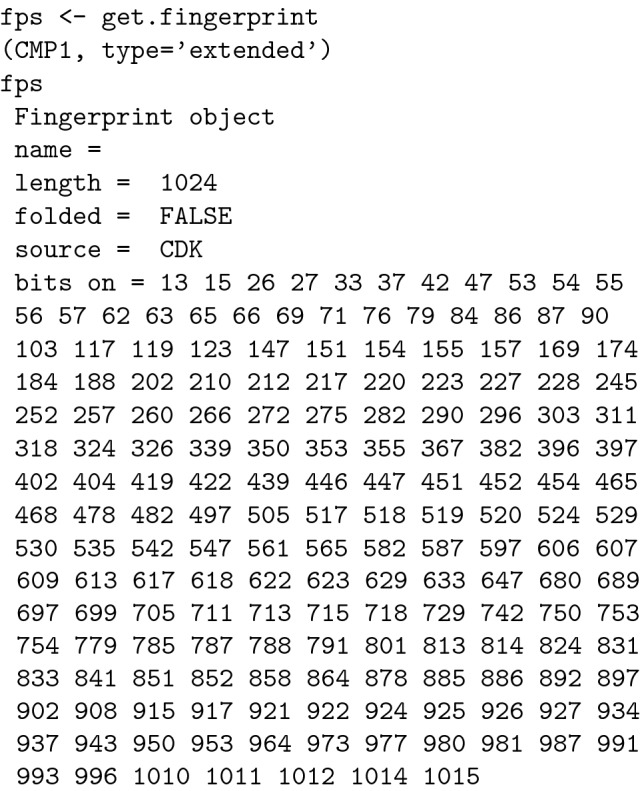


Similarly we computed the molecular fingerprints for the entire set of molecules: 
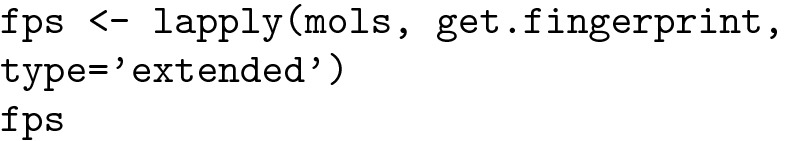


#### Computation of the intermolecular distances by the Tanimoto index

The Tanimoto coefficient can be expressed as:

$$\begin{aligned} S_{A,B}=c/[a+b-c] \end{aligned}$$

where S is the similarity, *a* is the number of on bits in molecule A, *b* is number of on bits in molecule B, and *c* is the number of on bits in both molecules [49].

Based on molecular fingerprints calculated using the Tanimoto method, the molecular distances between the methotrexate molecule and the other 23 molecules in the group can be evaluated: [24, 30]. 
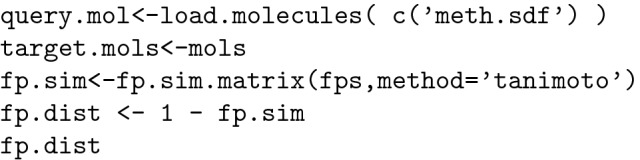


Using this method, a complete set of distances, in matrix form, between each of the 23 molecules of interest was obtained. By analysing these results, it is possible to identify all the molecules located at a certain distance from the target molecule (0.5 in our example): [48]. 
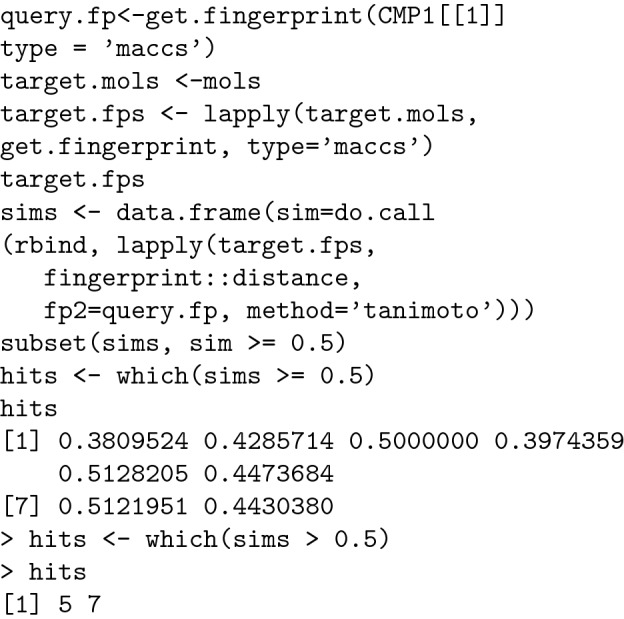


From the data presented above we can conclude that only molecules 5 and 7 meet our criteria. This method is the basis for fingerprint-based clustering.

## Results and Discussion

In the present study, we used a group of 23 newly synthesized molecules. All of them share the following characteristics: they are pyrazole derivatives, that have never been synthesized, there is no data about them in the literature or in chemical databases, and they have the potential to be drug candidates, such as purine derivatives. Our intention was to check whether the studied chemical compounds can be considered as possible lead molecules [26]. Because the costs of clinical trials are high, even in the preclinical phase, pre-sorting these candidates by computational chemistry and cheminformatics methods would be beneficial [2, 45]. According to the similarity property principle (SPP), which says that drugs with similar molecular structures are likely to have the same properties, a new drug candidate can be identified upon its similarity with another known drug, regardless of how the similarity is evaluated [5]. As a screening criterion, we used the comparison with the traditional methotrexate molecule [32].

*Methotrexate* is a cytotoxic substance widely used in cancer therapy. It was one of the first purine-inhibiting antimetabolites on the market, and it interferes with the growth of different molecules present in human body, such as like highly reproductive cancer cells. Even though this molecule cannot be considered a "gold standard" for this compound class, the above arguments have contributed to this choice.

### Clustering the dataset of molecules

Different types of methods can be used for clustering, including partitioning methods (K-means), hierarchical clustering, fuzzy clustering, density-based clustering and model-based clustering. The K-means and hierarchical clustering were chosen because they are suitable for our goal [29].

#### The number of clusters

**Fig. 3:**
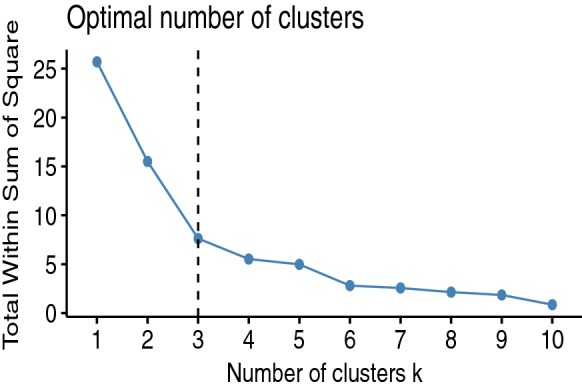
Estimation of the optimal number of clusters

The optimal number of clusters can be estimated using the NbClust package. The function fviz.nbclust is used for visualizing the result [1]. The R code for the elbow method is presented below: 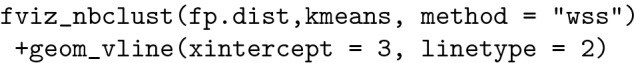


The result is depicted in Fig. 3:

The optimal number of clusters is 3.

All the considered molecules were then grouped into clusters by taking into account the calculated intermolecular distances.

#### Hierarchical clustering with the hclust package

The hierarchical clustering algorithm creates clusters with sets of data that are similar internally but different from each other externally [30]. The most common and useful graphical representation of molecular clusters is hierarchical clustering (dendrogram). We performed hierarchical clusterization using Ward’s method [30, 37].

Ward’s method is based on an ANOVA approach and its goal is to maximize the $r^{2}$ value.

**Fig. 4:**
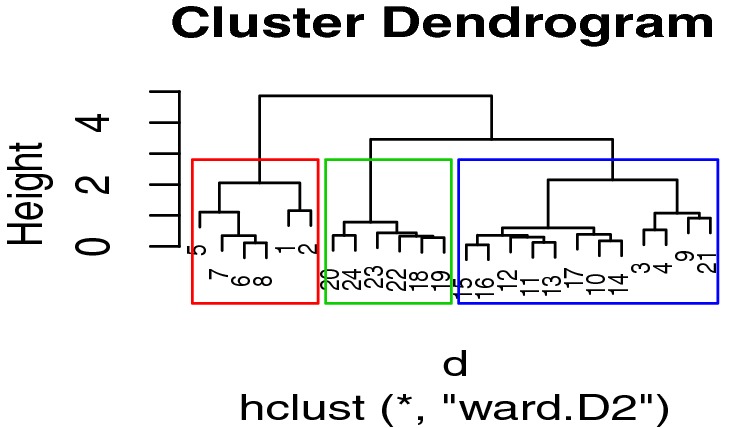
Dendrogram−hierarchical clustering using Ward’s method

To obtain this dendogram, we used the following R code: 
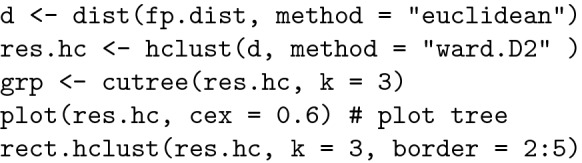


The graphical representation of the dendrogram is depicted in Fig. 4.

#### K-means Clustering

K-means clustering is one of the most commonly used clustering algorithms because it is easy to code and implement. Each cluster has a centre, which is called a centroid. The algorithm combines the distances between points and centroids [27]. The R code for K-means clustering is shown below. 
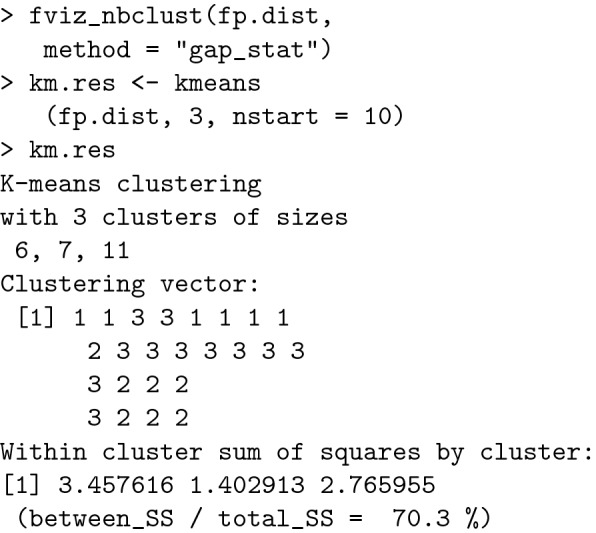


**Fig. 5:**
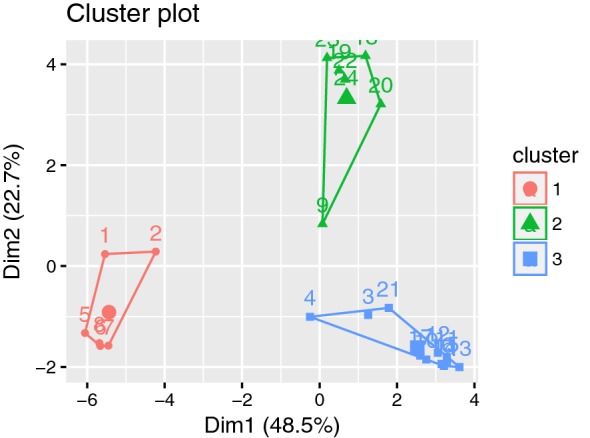
Polygonal clusters

**Fig. 6:**
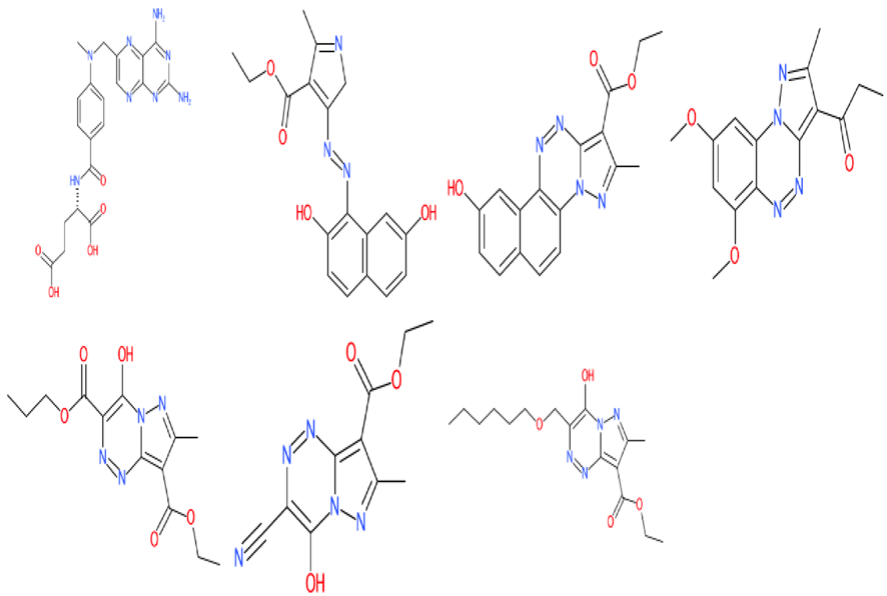
Molecules similar to methotrexate

The result is presented in Fig. 5:

The molecules included in cluster 1, containing methotrexate (CMP1), were visualized using the following R code: 
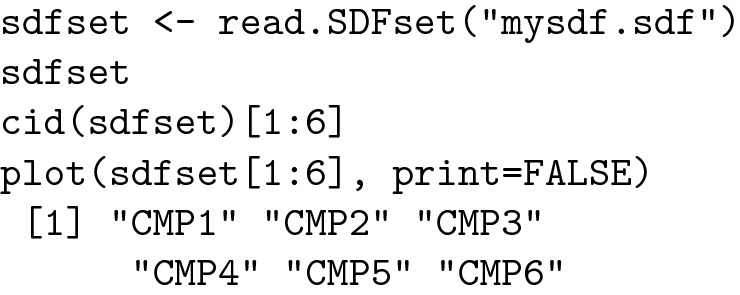


The result are depicted in Fig. 6:

#### Clustering validation

*Statistics for K-means clustering*

The R code for the cluster statistics is listed below: 
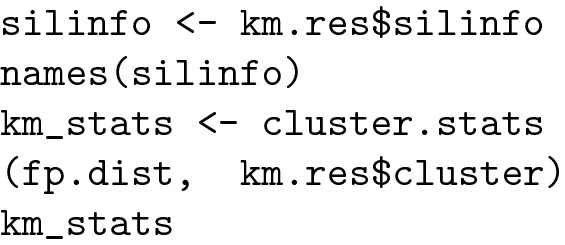


The most important information for the cluster analysis provided by this function can be considered the silhouette index and Dunn index: [7, 34]. 
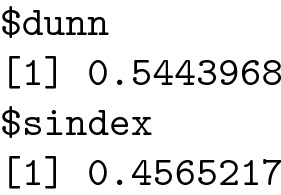


The Dunn index is equal to the ratio of the smallest inter-cluster distance divided by the largest intra-cluster distance [20].

It takes a value between zero and infinity, and a higher DI means that clusters are compact and well separated. A larger distance between clusters means a better separation, and smaller cluster sizes lead to a higher Dunn Index [1, 21].

The Silhouette coefficient is a method of cluster validation that combines both cohesion and separation [35]. It measures, for each point $M_{i}$, the mean distance to each cluster, and the mean distance to the other points in its cluster. Silhouette values range between − 1 and 1 [4]. A Silhouette coefficient with a value near +1 indicates that the point is far from its neighbouring cluster and very close to the cluster to which it is assigned. These values are preferred.

**Fig. 7:**
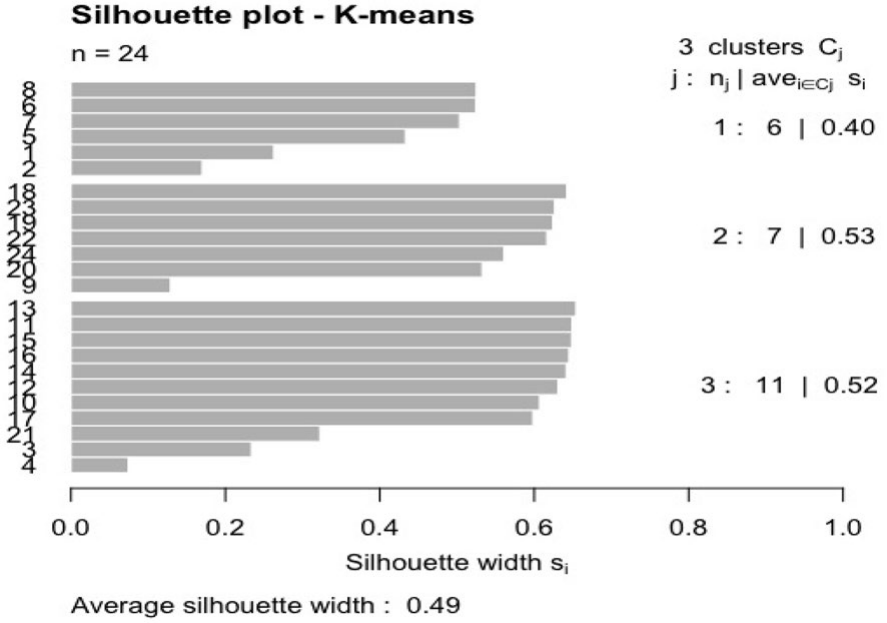
Silhouette plot for K-means clustering

The R code for visualizing a Silhouette plot for K-means clustering: 
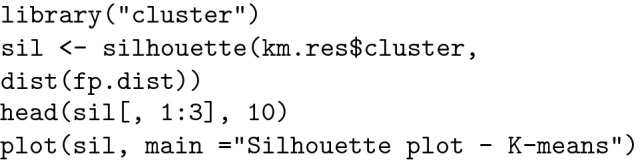


The plot is visualized in Fig. 7:

The Silhouette plot for K-means clustering reveals a coefficient of 0.4 for the first cluster and a mean value of 0.49 for all the other clusters. These values can be considered acceptable.

## Conclusions

Cheminformatics is a dynamic and powerful field that is considered the heart of modern drug design [9, 15]. It plays an important role in collecting, storing and analysing chemical data [18]. It is also an emerging interdisciplinary field that aims to discover new chemical entities that ultimately result in the design of a new active molecule (chemical data) [14, 22].

This work focused on cheminformatics and its application in the discovery and testing of new active molecules. In addition we focused on modern data mining techniques that help chemists and medical researchers to discover, produce and test new active molecules for the treatment of certain diseases.

Our goal was to test in vitro a set of 23 newly synthesized molecules, about which we do not have enough experimental data. The lack of information about the physicochemical properties of the respective molecules, especially those related to QSAR, was supplemented by the computational methods offered by the *rcdk* software package [12, 25].

Because we studied 23 compounds from the pyrazole class, we expected that at least some of them would behave similar to the like cytotoxic antimetabolite class (purine inhibitors). The clusters were obtained using hierarchical and K-means clustering methods. The results of clustering were confirmed using the Dunn index and Silhouette coefficient.

To avoid the additional costs that pre-clinical and clinical trials for all these compounds would have involved, we tried to reduce the number of "candidate drugs" by computational methods. This reduction was accomplished by calculating the molecular fingerprints of all the studied molecules and then comparing them with the molecular marker methotrexate, which still has a wide use. As a result of this comparison and after the clusterization of the molecules according to the Tanimoto distances, an optimal number of 3 clusters was obtained. In the cluster containing the methotrexate molecule of, marked with a 1, we can also find the molecules marked with a 2, 5, 6 and 7. The remaining 17 molecules are part of the other two clusters [30]. Therefore, starting from the assumption that "similar chemical structures have similar biological properties and actions", the number of compounds worth considering for further studies has been significantly reduced, from 23 to 4, which will lead to a significant decrease in all future costs [9].

## Data Availability

Data and materials are available on GitHub: The R code used for this paper: clusterch.R https://github.com/voicuadr/RClusters/blob/master/clusterch.R The cemical structures in sdf file format: CMP.sdf https://github.com/voicuadr/RClusters/blob/master/CMP.sdf
